# IntFOLD: an integrated web resource for high performance protein structure and function prediction

**DOI:** 10.1093/nar/gkz322

**Published:** 2019-05-02

**Authors:** Liam J McGuffin, Recep Adiyaman, Ali H A Maghrabi, Ahmad N Shuid, Danielle A Brackenridge, John O Nealon, Limcy S Philomina

**Affiliations:** 1 School of Biological Sciences, University of Reading, Whiteknights, Reading RG6 6AS, UK; 2 Infectomics cluster, Advanced Medical and Dental Institute, University of Science, Malaysia, Bertam, 13200, Kepala Batas, Pulau Pinang, Malaysia

## Abstract

The IntFOLD server provides a unified resource for the automated prediction of: protein tertiary structures with built-in estimates of model accuracy (EMA), protein structural domain boundaries, natively unstructured or disordered regions in proteins, and protein–ligand interactions. The component methods have been independently evaluated via the successive blind CASP experiments and the continual CAMEO benchmarking project. The IntFOLD server has established its ranking as one of the best performing publicly available servers, based on independent official evaluation metrics. Here, we describe significant updates to the server back end, where we have focused on performance improvements in tertiary structure predictions, in terms of global 3D model quality and accuracy self-estimates (ASE), which we achieve using our newly improved ModFOLD7_rank algorithm. We also report on various upgrades to the front end including: a streamlined submission process, enhanced visualization of models, new confidence scores for ranking, and links for accessing all annotated model data. Furthermore, we now include an option for users to submit selected models for further refinement via convenient push buttons. The IntFOLD server is freely available at: http://www.reading.ac.uk/bioinf/IntFOLD/.

## INTRODUCTION

Despite recent advances in the experimental methods for determining protein tertiary structures and their interactions, the sequence-to-structure gap has been relentlessly increasing. The gap in our knowledge of protein sequences versus known structures is being exacerbated by onset of ever cheaper and more efficient genome sequencing methods. At the time of writing, we now have close to two hundred million unique protein sequences in UniProt (1), but the number of protein structures in the Protein Data Bank (PDB) (2) remains <150 000. In order to realize the promise of next generation sequencing, it is clear that we must rely on computational tools for predicting structures and building 3D models of proteins directly from sequence so that we may close the knowledge gap. While the routine use of predicted 3D models by life scientists continues to grow, the protein structure prediction community has faced a number of challenges, which may have restricted the more wide spread acceptance of 3D protein models by non-experts (3). For example, until relatively recently we have not had methods that can confidently estimate the likely quality of 3D protein models, although these tools are now becoming increasingly accurate and more widely available (4).

The structure prediction community has made great advances over the past 20+ years with several major improvements in template based modelling (TBM), free modelling (FM) and estimates of 3D model accuracy (EMA) coming in the last few CASP (Critical Assessment of Structure Prediction) experiments (5–7). Successive versions of the IntFOLD server components have been independently benchmarked in the CASP experiments, from CASP9 to CASP13, and continually by the CAMEO project (8). Many of our own advances in performance over the years have come through improvements in our ModFOLD methods for EMA, and in particular our Accuracy Self Estimate (ASE) scoring for our 3D models (5,9).

Previous versions of the IntFOLD server were described in the Web Server issues of this journal in 2011 (10) and 2015 (11). Since its inception, the server has had ∼15,000 unique users and it has completed ∼200 000 predictions. The server's component methods have been applied in order to model protein structures and their interactions for a diverse range of specialisations accross the life sciences. For example, our tools have been used: to model novel proteins in the *Drosophila melanogaster* genome (12), to reveal new interactions and mechanisms for the regulation of mammalian GCKIII kinases (13,14), to explain the evolutionary resurrection of flagellar motility in *Pseudomonas fluorescens* (15), to structurally and functionally annotate the proteome of barley powdery mildew (*Blumeria graminis* f. sp. *hordei*) (16), and to understand the effect of the missense mutation associated with dermatosparaxis (17).

In this paper, we describe the significant modifications to IntFOLD server and its component methods, which have led to successive performance gains since our last paper on the server from 2015. As well as reporting the major enhancements ‘under the hood’ to the server backend, we also report on the provision of new data outputs and user interface improvements.

## MATERIALS AND METHODS

The IntFOLD server provides a single point of access to an integrated suite of six component methods: IntFOLD-TS, for tertiary structure prediction (9–11,18,19); ModFOLD, for 3D model Accuracy Self-Estimate (ASE) scoring (9,20); ReFOLD, for 3D model refinement (9,21); DISOclust, for disorder prediction (22,23); DomFOLD for structural domain prediction (10,11) and FunFOLD for ligand binding site prediction (24,25). These component methods have been independently evaluated in the various CASP (5,7,26–28) experiments over the years and are continually benchmarked by the CAMEO project (8) (also see results section). The major enhancement to the server methodology, since the last web server paper, has been to the underlying Tertiary Structure (TS) prediction algorithm. Since its inception, the high performance tertiary structure prediction algorithms with integrated model quality assessment have been at the core of IntFOLD server (10,11,18), and these factors have been key contributors to the historical success of the component methods (5,7,9,18,26–30). For version 5 of the IntFOLD server, the algorithms for both 3D model selection and ASE scoring have been upgraded via the integration of our new ModFOLD7_rank method.

The IntFOLD-TS method is the major component of the server and its output of high quality 3D models forms the basis for subsequent prediction algorithms. The IntFOLD5-TS method was newly developed for CASP13 and worked via iterative multi-template based modelling (19) using the target-template alignments from 14 alternative methods (SP3 (31), SPARKS2 (31), HHsearch (32), COMA (33), SPARKSX (34), CNFsearch (35) and the eight alternative threading methods that are integrated into the current LOMETS package (36)). The multiple target-template alignments for 3D modelling were then selected using ASE scoring via the ModFOLD7_rank method, with the aim of minimising local errors in final generated models. Additionally, the HHpred (37) method and the template free method I-TASSER light (38) (for sequence <500 residues; run in ‘light mode’ with wall-time restricted to 5h) contributed models for ranking. All of the final models were pooled and then scored and ranked using the ModFOLD7_rank method and presented to the user in descending order of global model quality. The ASE scores from ModFOLD7_rank were included in the temperature factor column of each of the PDB formatted model files. The integration of ASE scores in this way allows users to conveniently view the local model quality as temperature gradient that can be mapped onto their 3D models using their favourite molecular viewing software, for example PyMOL (http://www.pymol.org/).

The ModFOLD7_rank method is our latest update to Quality Assessment (QA) that combines the strengths of multiple pure-single and quasi-single model methods for improving prediction accuracy, building on the successful strategy that was used in ModFOLD6 (4,9,20). For the IntFOLD5 server our major emphasis was on increasing the performance of per-residue accuracy prediction for our own models, as well as improving our model ranking and score consistency for our models. Each IntFOLD5 model was considered individually using 6 pure-single model methods (CDA (20), SSA (20), ProQ2 (4), ProQ2D (39), ProQ3D (39) and VoroMQA (40)), and four alternative quasi-single model methods (DBA (20), MF5s (20), MFcQs (20) and ResQ (41)). For producing final local score outputs, Artificial Neural networks (NNs) were used to combine the component per-residue/local quality scores from each of the 10 alternative scoring methods, resulting in a final consensus of per-residue quality scores for each model. For producing the global score outputs, we made several variants that combined the mean global scores from the different methods and each were optimized for different aspects of the quality estimation problem. For the IntFOLD5 server, the accurate ranking of our models was the main objective, so for this reason we integrated the ModFOLD7_rank variant, which was optimized for ranking.

As well as improvements in performance to underlying algorithms, several new user interface upgrades were implemented. These included a streamlined submission form, recalibrated *P*-values for confidence scoring of model quality estimates, the ability to download compressed archives of all annotated models, and the ability to interact with models and then further refine them with a few clicks via simple push buttons. The server inputs and outputs are described in more detail below.

## RESULTS AND DISCUSSION

### Server inputs and outputs

#### Inputs

A single amino acid sequence for the protein chain is the only required input for the server. However, users also have the option to provide a short memorable name for their prediction job and an email address, which will only be used to provide a notification of the link to the results when the predictions are completed. If users do not wish to be notified via email, then they can bookmark the link to the results page for later viewing.

#### Graphical outputs

**Figure 1.**
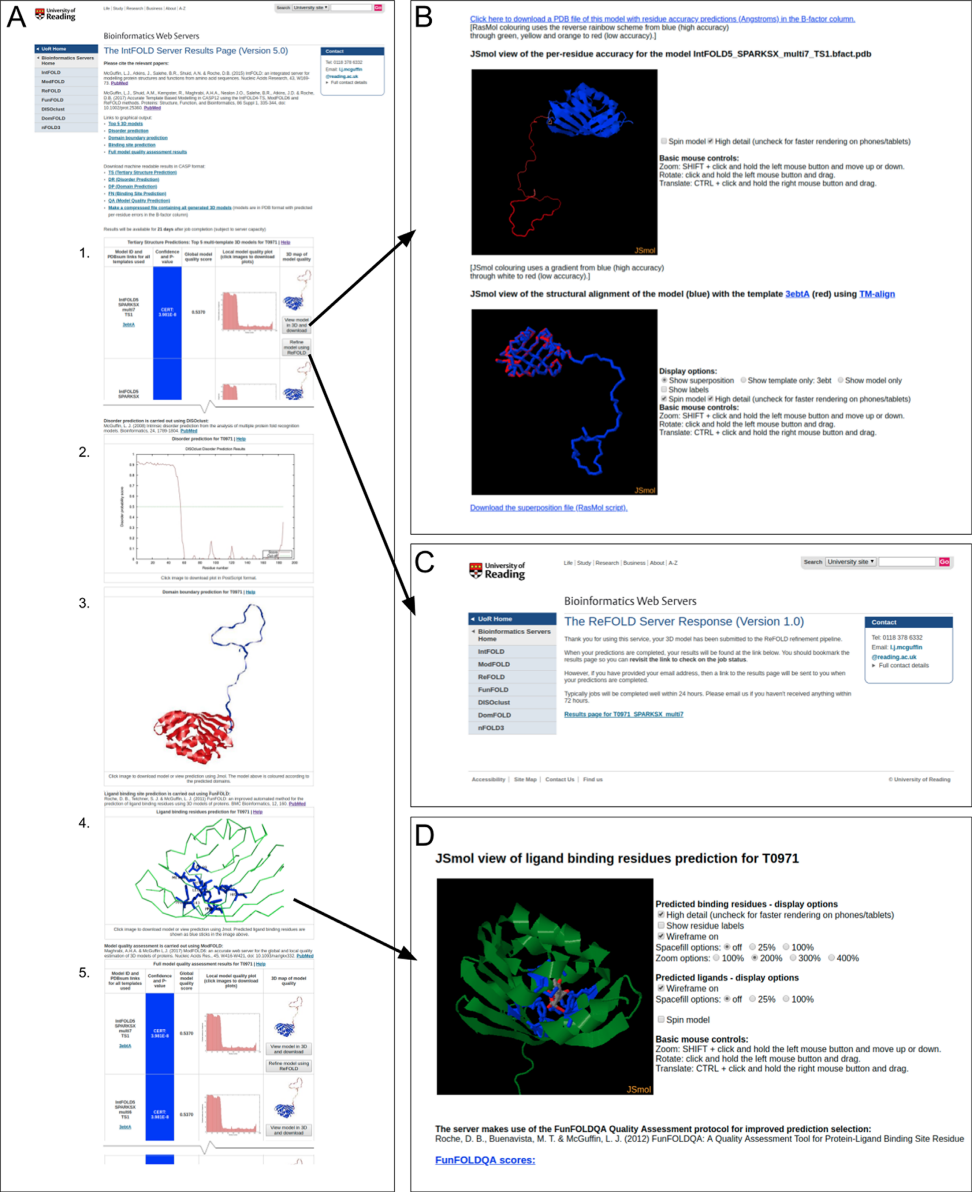
The IntFOLD5 server results pages for CASP13 target T0971. (**A**) Graphical output from the main results page showing (from top to bottom): 1. The table with the top 5 selected 3D models and scores (table truncated here to fit); 2. The prediction of natively unstructured/disordered regions; 3. The predicted structural domain boundaries; 4. The ligand binding site prediction; 5. The full model quality rankings for all generated models (table truncated here to fit). The arrows point to additional pages that are linked to when users click on images/buttons on the main page. (**B**) Clicking the button titled ‘View model in 3D and download’ leads to dynamically generated pages showing interactive views of the model, and structural superpositions of the model with relevant template/s, which can be manipulated in 3D using the JSmol/HTML5 framework (http://www.jmol.org/) and/or downloaded for local viewing. (**C**) Clicking the button titled ‘Refine model using ReFOLD’ submits the 3D model to the ReFOLD service (21) for refinement guided by accurate quality estimates. (**D**) Clicking on the image of the ligand binding site prediction links to a dynamically generated page that provides numerous options for interactively viewing the likely protein–ligand interactions in 3D with JSmol.

Examples of the graphical outputs from the IntFOLD5 server are shown in Figure 1. The graphical output is presented as a single table that graphically summarises all prediction data using thumbnail images of ASE plots and models, links to the template information and colour coded scoring (Figure 1A). It is always recommended to choose the model with the highest score or lowest *P*-value. The confidence rating relates to the *P*-value. For example, a ‘CERT’ rating relates to models where *P* < 0.001, i.e., less than a 1/1000 chance that the model is incorrect (see help pages for other ratings). So all ‘CERT’ models are highly likely to have the correct fold. However, the models with the lowest *P*-values are more likely to have the highest backbone accuracy and overall quality. Several new user interface options are available. Users have the option to download coordinates and view the detailed IntFOLD5-TS tertiary structure prediction results interactively in 3D (Figure 1B) and submit individual 3D models for further refinement using ReFOLD (Figure 1C) via simple push buttons. Downloadable coordinates and interactive 3D views of the protein ligand interactions can also be accessed via the FunFOLD results summary image (Figure 1D). In addition, clicking on the DISOclust disorder prediction profile images and the thumbnail images of the ASE score profiles from ModFOLD7_rank will allow users to view and/or download higher quality versions of the plots.

**Figure 2.**
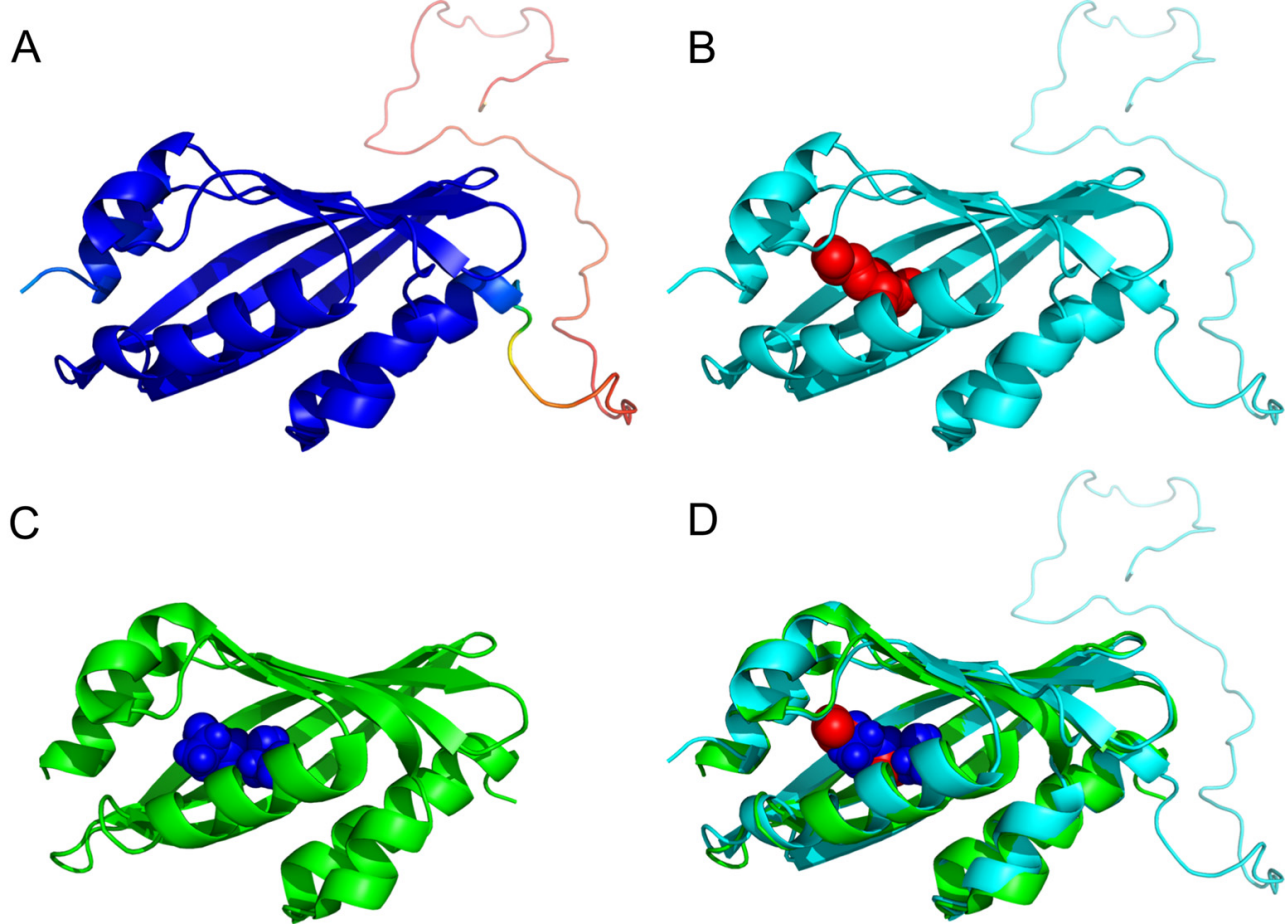
The IntFOLD5 server predictions for CASP13 target T0971 – comparison of models with the native crystal structure (PDB ID: 6d34). All images were rendered using PyMOL (http://www.pymol.org/). (**A**) The IntFOLD5 3D model coloured by accuracy self-estimate of local quality using the temperature coloured scheme from blue (indicating residues in the model predicted to be close to the native structure) to red (indicating residues in the model that are far from the native or unstructured). (**B**) The IntFOLD5 3D model with the main cluster of predicted ligands (red spheres) indicating the predicted location of binding site. (**C**) The crystal structure of T0971/6d34 with ligand (blue spheres). Note: the disordered domain predicted in the model is absent in the X-ray data. (**D**) Superposition of the IntFOLD5 model and the native structure.

Figure 2 shows a comparison of the example models for CASP13 target T0971 (obtained via the pages shown in Figure 1) and the native structure (PDB ID 6d34). The 3D model of the protein (Figure 2A and B) is close to the native structure shown in Figure 2C. The predicted location of the ligand binding site is shown to be accurate (Figure 2B) and there is a close superposition of the model and native structure (Figure 2D), with a GDT_TS score of 95%. The ASE for the model, indicated by the colouring in Figure 2A, and the identification of the unstructured domain are also shown to be accurately predicted.

#### Machine readable outputs

All of the raw data files for the predictions are available to download via links on the results pages. The file formats comply with the CASP and/or CAMEO data standards. An additional new feature is the provision of a link that allows users to download all of the ASE annotated models in PDB format (with the error estimates, in Angstroms, in place of temperature factor data) as a zipped archive.

### Independent benchmarking

Each major version of the server has been independently tested in each of the relevant categories of the CASP experiments (from CASP9 to CASP13, http://predictioncenter.org) and the performance has been competitive (9,18). Recently, the component methods have ranked among top independent servers in the Tertiary Structure (TS) prediction (5) and Estimates of Model Accuracy (EMA) categories (7), as well as ranking well in the historical categories of intrinsic disorder prediction and function prediction (26,27). The DISOclust method was designed to add a significant performance boost to DISOPRED (22), and the latest version of DISOPRED is integrated with the IntFOLD server. Additionally, the IntFOLD5 server components (IntFOLD, ModFOLD and FunFOLD) have been continuously benchmarked using the CAMEO resource (8) and they have been shown to be competitive in each respective category (see results from the 3D, QE and LB categories at https://www.cameo3d.org/). Furthermore, the GO term outputs from the FunFOLD component of the server have been benchmarked during the most recent CAFA experiment (https://www.biofunctionprediction.org/cafa/, paper in preparation).

#### CAMEO results summary

The TS predictions from the IntFOLD5 server are continuously evaluated by the CAMEO project (8). The IntFOLD versions have consistently ranked among the top few public servers according to lDDT_BS scores and lDDT scores. At the time of writing, IntFOLD5-TS ranks as the top publicly available method based on the last 3-month data for all targets (Table 1). Based on pairwise comparisons using a common subset of targets over the last year, IntFOLD5-TS ranks as the second best 3D server according to the CAMEO lDDT scores (Supplementary Tables S1 and S2). Moreover, the IntFOLD5-TS version of the method has been independently verified to be an improvement over our two previous methods (IntFOLD3-TS and IntFOLD4-TS) (Table 2).

**Table 1.** Independent benchmarking of tertiary structure predictions with CAMEO 3D data. Performance results for 3 months of data (26 October 2018 to 19 January 2019) are shown for *all* (250) targets and *all* (17) public methods. Data are sorted by average lDDT score for all targets. The scores for the IntFOLD-TS methods are indicated in bold. Data are taken from the CAMEO 3D front page http://www.cameo3d.org/ on 19 January 2019.

	Average lDDT		Average lDDT-BS	
Server name	All targets	Modelled targets	All targets	Modelled targets
**IntFOLD5-TS**	**68.04**	**68.04**	**70.94**	**70.94**
RaptorX	67.38	67.38	68.45	68.45
Robetta	65.51	69.1	63.24	66.11
HHpredB	64.06	64.06	68.59	68.59
SWISS-MODEL	62.22	62.97	64.85	65.56
**IntFOLD4-TS**	**55.02**	**68.1**	**58.12**	**73.25**
SPARKS-X	54.63	60.7	58.07	66.78
M4T-SMOTIF-TF	54.45	60.77	62.92	65.78
**IntFOLD3-TS**	**53.75**	**66.85**	**55.76**	**69.33**
PRIMO	51.74	57.48	58.32	64.65
PRIMO_BST_CL	51.71	57.45	58.32	64.65
NaiveBLAST	50.34	55.69	60.08	62.11
PRIMO_BST_3D	49.83	55.86	57.99	63.51
PRIMO_HHS_3D	48.27	55.87	56.49	62.62
PRIMO_HHS_CL	46.73	56.43	55.55	61.58
Princeton_TEMPLATE	24.46	54.61	25.63	58.95
Phyre2	24.06	52.77	29.27	67.31

**Table 2.** Independent benchmarking of IntFOLD versions with CAMEO 3D data showing the sequential improvement in server performance since the last webserver paper describing IntFOLD3. Performance results for 1 year of data (26 January 2018 to 19 January 2019) are shown for a common subset of 581 targets. The reference method is IntFOLD5-TS and the table is sorted by average lDDT. Data are downloaded from http://www.cameo3d.org/

	Avg. lDDT		Avg. CAD-score		Avg. lDDT-BS	
Server Name	Dif.	Ref.	Dif.	Ref.	Dif.	Ref.
IntFOLD5-TS	0	67.72	0	0.67	0	71.86
IntFOLD4-TS	0.53	67.18	0	0.66	0.23	71.62
IntFOLD3-TS	2.11	65.61	0.02	0.65	1.9	69.96

#### CASP12 and 13 results summary

In the last few CASP experiments since the last webserver publication, the IntFOLD server has performed well at Template Based Modelling (TBM), ranking as high as third place and outperforming other servers in terms of Accuracy Self Estimates (ASE) (5). The IntFOLD4 and IntFOLD5 server performance rankings, for CASP12 and CASP13 targets respectively, are shown in Supplementary Tables S3–S6. The IntFOLD server methods have also been key to our group's success at CASP12 and 13 allowing us to rank as high as second place on the ‘all group’ TBM + TBM/FM domains. The McGuffin group performance is summarized in Supplementary Tables S7 and S8.

## CONCLUSIONS

The IntFOLD server provides free access to an integrated set of high performance, fully automated tools for structure and function prediction of proteins from their amino acid sequences. The component methods of the server are continually benchmarked via the CAMEO project and they have been rigorously blind tested at recent CASP experiments. The IntFOLD methods have been independently verified to rank among the top performing servers in many prediction categories. Results from the IntFOLD server are presented to non-expert users in an intuitive manner with graphical output providing a visual summary of a complex set of data. More detailed results for individual predictions can be interactively viewed and the raw, machine readable data can be accessed in standard data formats.

## Supplementary Material

gkz322_Supplemental_File
