# Supplementary material for: IntFOLD: an integrated web resource for high performance protein structure and function prediction
Source: Nucleic Acids Res. 2019 May 2;47(W1):W408–13. doi: 10.1093/nar/gkz322 (PMC6602432; doi:10.1093/nar/gkz322)
Supplement: gkz322_Supplemental_File [file gkz322_supplemental_file.docx]

**SUPPLEMENTARY TABLES**

Supplementary Table 1. Independent benchmarking of tertiary structure predictions with CAMEO 3D data. Performance results for 1 year of data (2018-01-26 to 2019-01-19) are shown for a common subset of 199 targets for *all* (17) public methods. The reference method is IntFOLD5-TS and the table is sorted by average lDDT. Data are downloaded from <http://www.cameo3d.org/>.

|  | **Avg.  lDDT** | | **Avg. CAD-score** | | **Avg. lDDT-BS** | |
| --- | --- | --- | --- | --- | --- | --- |
| **Server Name** | **Dif.** | **Ref.** | **Dif.** | **Ref.** | **Dif.** | **Ref.** |
| Robetta | -1.67 | 72.45 | -0.02 | 0.7 | 5.54 | 71.32 |
| **IntFOLD5-TS** | **0** | **70.78** | **0** | **0.69** | **0** | **76.85** |
| RaptorX | 0.18 | 70.6 | 0 | 0.68 | 4.84 | 72.01 |
| **IntFOLD4-TS** | **0.45** | **70.33** | **0** | **0.68** | **0.27** | **76.58** |
| **IntFOLD3-TS** | **1.82** | **68.96** | **0.02** | **0.67** | **1.43** | **75.42** |
| SWISS-MODEL | 2.64 | 68.14 | 0.03 | 0.66 | 0.47 | 76.38 |
| HHpredB | 3.64 | 67.14 | 0.01 | 0.67 | 6.26 | 70.6 |
| M4T-SMOTIF-TF | 5.46 | 65.32 | 0.05 | 0.64 | 1.79 | 75.07 |
| SPARKS-X | 6.39 | 64.38 | 0.04 | 0.64 | 5.94 | 70.92 |
| PRIMO | 7.17 | 63.61 | 0.06 | 0.63 | 5.02 | 71.83 |
| PRIMO_BST_CL | 7.17 | 63.61 | 0.06 | 0.63 | 5.02 | 71.83 |
| PRIMO_BST_3D | 8.47 | 62.3 | 0.07 | 0.61 | 6.92 | 69.93 |
| PRIMO_HHS_3D | 8.86 | 61.92 | 0.08 | 0.61 | 6.95 | 69.91 |
| PRIMO_HHS_CL | 9.31 | 61.47 | 0.08 | 0.61 | 9.26 | 67.59 |
| NaiveBLAST | 9.74 | 61.04 | 0.1 | 0.59 | 4.37 | 72.49 |
| Princeton_TEMPLATE | 10.26 | 60.52 | 0.09 | 0.59 | 18.63 | 58.22 |
| Phyre2 | 13.15 | 57.62 | 0.06 | 0.63 | 4.74 | 72.12 |

Supplementary Table 2. Independent benchmarking of tertiary structure predictions with CAMEO 3D data. Performance results for 1 year of data (2018-01-26 to 2019-01-19) are shown for a common subset of 575 targets for the top 3 public methods plus the older versions of IntFOLD. The reference method is IntFOLD5-TS and the table is sorted by average lDDT. Data are downloaded from <http://www.cameo3d.org/>.

|  | **Avg.  lDDT** | | **Avg. CAD-score** | | **Avg. lDDT-BS** | |
| --- | --- | --- | --- | --- | --- | --- |
| **Server Name** | **Dif.** | **Ref.** | **Dif.** | **Ref.** | **Dif.** | **Ref.** |
| Robetta | -1.32 | 69.07 | -0.02 | 0.68 | 5.8 | 66 |
| **IntFOLD5-TS** | **0** | **67.75** | **0** | **0.67** | **0** | **71.81** |
| **IntFOLD4-TS** | **0.52** | **67.23** | **0** | **0.66** | **0.24** | **71.57** |
| RaptorX | 0.58 | 67.17 | 0 | 0.66 | 4.85 | 66.95 |
| **IntFOLD3-TS** | **2.1** | **65.65** | **0.02** | **0.65** | **1.9** | **69.9** |

Supplementary Table 3. Independent benchmarking or tertiary structure predictions with CASP12 data. “Assessors’ formula” (GDT_HA + (SG + lDDT + CAD)/3 + ASE) data are shown for the server groups on the 'all groups' + 'server only' targets for the TBM + TBM/FM domains. Data are downloaded from <http://predictioncenter.org/casp12/>.

| **#** | **GR code** | **GR name** | **Domains Count** | **SUM Zscore (>-2.0)** | **Rank SUM Zscore (>-2.0)** | **AVG Zscore (>-2.0)** | **Rank AVG Zscore (>-2.0)** | **SUM Zscore (>0.0)** | **Rank SUM Zscore (>0.0)** | **AVG Zscore (>0.0)** | **Rank AVG Zscore (>0.0)** |
| --- | --- | --- | --- | --- | --- | --- | --- | --- | --- | --- | --- |
| 1 | 479 | Zhang-Server | 57 | 53.2687 | 1 | 0.9345 | 1 | 53.558 | 1 | 0.9396 | 1 |
| 2 | 183 | QUARK | 57 | 51.2984 | 2 | 0.9 | 2 | 51.8455 | 2 | 0.9096 | 2 |
| **3** | **405** | **IntFOLD4** | **57** | **38.1512** | **3** | **0.6693** | **3** | **40.7005** | **5** | **0.714** | **5** |
| 4 | 250 | Seok-server | 57 | 36.5368 | 4 | 0.641 | 4 | 41.7567 | 4 | 0.7326 | 4 |
| 5 | 236 | MULTICOM-CONSTRUCT | 57 | 34.5974 | 5 | 0.607 | 5 | 37.0262 | 8 | 0.6496 | 8 |
| 6 | 287 | MULTICOM-CLUSTER | 57 | 34.3201 | 6 | 0.6021 | 6 | 37.661 | 7 | 0.6607 | 7 |
| 7 | 345 | MULTICOM-NOVEL | 57 | 29.3235 | 7 | 0.5144 | 8 | 33.4039 | 9 | 0.586 | 10 |
| 8 | 220 | GOAL | 57 | 24.4642 | 8 | 0.4292 | 9 | 40.0205 | 6 | 0.7021 | 6 |
| 9 | 5 | BAKER-ROSETTASERVER | 57 | 22.6251 | 9 | 0.3969 | 10 | 43.5567 | 3 | 0.7642 | 3 |
| 10 | 119 | HHPred0 | 57 | 10.6627 | 10 | 0.1871 | 11 | 21.3322 | 14 | 0.3742 | 17 |
| 11 | 349 | HHPred1 | 57 | 10.2547 | 11 | 0.1799 | 12 | 21.102 | 15 | 0.3702 | 18 |
| 12 | 251 | myprotein-me | 57 | 7.6733 | 12 | 0.1346 | 13 | 25.0498 | 12 | 0.4395 | 13 |
| 13 | 48 | ToyPred_email | 57 | 6.0518 | 13 | 0.1062 | 14 | 27.6669 | 10 | 0.4854 | 11 |
| 14 | 92 | RaptorX | 57 | 5.921 | 14 | 0.1039 | 15 | 27.6238 | 11 | 0.4846 | 12 |
| 15 | 313 | HHGG | 57 | 4.1865 | 15 | 0.0734 | 16 | 24.6569 | 13 | 0.4326 | 14 |
| 16 | 425 | FALCON_TOPOX | 57 | -5.3579 | 16 | -0.094 | 18 | 16.0002 | 21 | 0.2807 | 23 |
| 17 | 77 | FALCON_TOPO | 57 | -5.3673 | 17 | -0.0942 | 19 | 16.4832 | 19 | 0.2892 | 21 |
| 18 | 16 | FFAS-3D | 57 | -7.8423 | 18 | -0.1376 | 21 | 14.3766 | 23 | 0.2522 | 26 |
| 19 | 446 | YASARA | 56 | -8.1083 | 19 | -0.1091 | 20 | 20.5859 | 17 | 0.3676 | 19 |
| 20 | 464 | tsspred2 | 57 | -10.2517 | 20 | -0.1799 | 22 | 12.695 | 27 | 0.2227 | 30 |
| 21 | 407 | Distill | 56 | -16.1758 | 21 | -0.2531 | 23 | 10.9687 | 31 | 0.1959 | 34 |
| 22 | 258 | MUfold1 | 57 | -18.1764 | 22 | -0.3189 | 26 | 13.2322 | 26 | 0.2321 | 28 |
| 23 | 452 | ZHOU-SPARKS-X | 52 | -23.3181 | 23 | -0.2561 | 24 | 13.3672 | 25 | 0.2571 | 25 |
| 24 | 166 | FFAS03 | 53 | -23.7933 | 24 | -0.298 | 25 | 10.4745 | 32 | 0.1976 | 33 |
| 25 | 467 | Pareto-server | 55 | -24.2596 | 25 | -0.3684 | 28 | 11.2497 | 30 | 0.2045 | 32 |
| 26 | 359 | Atome2_CBS | 53 | -25.5309 | 26 | -0.3308 | 27 | 12.06 | 28 | 0.2275 | 29 |
| 27 | 382 | RBO_Aleph | 57 | -29.2388 | 27 | -0.513 | 30 | 16.2245 | 20 | 0.2846 | 22 |
| 28 | 444 | BhageerathH-Plus | 57 | -33.0442 | 28 | -0.5797 | 32 | 10.2702 | 33 | 0.1802 | 35 |
| 29 | 357 | FLOUDAS_SERVER | 56 | -33.4333 | 29 | -0.5613 | 31 | 9.4264 | 34 | 0.1683 | 36 |
| 30 | 434 | MULTICOM-REFINE | 57 | -36.894 | 30 | -0.6473 | 33 | 16.6907 | 18 | 0.2928 | 20 |
| 31 | 275 | slbio | 57 | -39.6457 | 31 | -0.6955 | 36 | 13.5094 | 24 | 0.237 | 27 |
| 32 | 455 | ACOMPMOD | 53 | -43.8602 | 32 | -0.6766 | 34 | 20.7454 | 16 | 0.3914 | 16 |
| 33 | 28 | M4T-SmotifTF | 46 | -44.1226 | 33 | -0.4809 | 29 | 11.8749 | 29 | 0.2581 | 24 |
| 34 | 495 | Seok-assembly | 26 | -48.4298 | 34 | 0.5219 | 7 | 15.4743 | 22 | 0.5952 | 9 |
| 35 | 26 | chuo-u2 | 57 | -55.1508 | 35 | -0.9676 | 37 | 3.7104 | 39 | 0.0651 | 40 |
| 36 | 380 | chuo-u-server | 57 | -55.1508 | 35 | -0.9676 | 37 | 3.7104 | 39 | 0.0651 | 40 |
| 37 | 180 | PhyreTopoAlpha | 57 | -55.9052 | 37 | -0.9808 | 39 | 5.5023 | 38 | 0.0965 | 39 |
| 38 | 421 | MUfold2 | 53 | -61.5494 | 38 | -1.0104 | 40 | 5.7968 | 36 | 0.1094 | 38 |
| 39 | 451 | RaptorX-Contact | 55 | -72.8008 | 39 | -1.2509 | 42 | 3.3273 | 41 | 0.0605 | 42 |
| 40 | 432 | Pcons-net | 47 | -76.9522 | 40 | -1.2117 | 41 | 7.786 | 35 | 0.1657 | 37 |
| 41 | 321 | GAPF_LNCC_SERVER | 55 | -80.1587 | 41 | -1.3847 | 43 | 1.1581 | 43 | 0.0211 | 43 |
| 42 | 430 | GOAL_COMPLEX | 13 | -88.2172 | 42 | -0.0167 | 17 | 5.6219 | 37 | 0.4325 | 15 |
| 43 | 284 | Seok-naive_assembly | 15 | -94.3579 | 43 | -0.6905 | 35 | 3.2271 | 42 | 0.2151 | 31 |

Supplementary Table 4. Independent benchmarking or tertiary structure predictions with CASP12 data. “Assessors’ formula” (GDT_HA + (SG + lDDT + CAD)/3 + ASE) data are shown for the server groups on the 'all groups' + 'server only' targets for the TBM domains only. Data are downloaded from <http://predictioncenter.org/casp12/>.

| **#** | **GR code** | **GR name** | **Domains Count** | **SUM Zscore (>-2.0)** | **Rank SUM Zscore (>-2.0)** | **AVG Zscore (>-2.0)** | **Rank AVG Zscore (>-2.0)** | **SUM Zscore (>0.0)** | **Rank SUM Zscore (>0.0)** | **AVG Zscore (>0.0)** | **Rank AVG Zscore (>0.0)** |
| --- | --- | --- | --- | --- | --- | --- | --- | --- | --- | --- | --- |
| 1 | 479 | Zhang-Server | 38 | 33.6924 | 1 | 0.8866 | 1 | 33.7571 | 1 | 0.8883 | 1 |
| 2 | 183 | QUARK | 38 | 32.5966 | 2 | 0.8578 | 2 | 32.7542 | 2 | 0.862 | 2 |
| 3 | 250 | Seok-server | 38 | 27.4052 | 3 | 0.7212 | 3 | 29.6166 | 3 | 0.7794 | 3 |
| **4** | **405** | **IntFOLD4** | **38** | **26.0296** | **4** | **0.685** | **4** | **27.6067** | **5** | **0.7265** | **5** |
| 5 | 287 | MULTICOM-CLUSTER | 38 | 25.7573 | 5 | 0.6778 | 5 | 26.332 | 7 | 0.6929 | 7 |
| 6 | 236 | MULTICOM-CONSTRUCT | 38 | 25.7494 | 6 | 0.6776 | 6 | 26.4802 | 6 | 0.6968 | 6 |
| 7 | 345 | MULTICOM-NOVEL | 38 | 20.7421 | 7 | 0.5458 | 8 | 22.3422 | 9 | 0.588 | 10 |
| 8 | 5 | BAKER-ROSETTASERVER | 38 | 17.4594 | 8 | 0.4595 | 9 | 29.3119 | 4 | 0.7714 | 4 |
| 9 | 220 | GOAL | 38 | 14.6972 | 9 | 0.3868 | 10 | 25.6018 | 8 | 0.6737 | 8 |
| 10 | 349 | HHPred1 | 38 | 9.8043 | 10 | 0.258 | 11 | 13.8702 | 15 | 0.365 | 17 |
| 11 | 119 | HHPred0 | 38 | 9.7293 | 11 | 0.256 | 12 | 13.7582 | 16 | 0.3621 | 18 |
| 12 | 251 | myprotein-me | 38 | 8.2083 | 12 | 0.216 | 14 | 18.7389 | 10 | 0.4931 | 12 |
| 13 | 446 | YASARA | 37 | 6.2872 | 13 | 0.224 | 13 | 18.2826 | 11 | 0.4941 | 11 |
| 14 | 313 | HHGG | 38 | 3.0155 | 14 | 0.0794 | 15 | 15.9226 | 14 | 0.419 | 16 |
| 15 | 48 | ToyPred_email | 38 | 2.576 | 15 | 0.0678 | 16 | 17.0001 | 12 | 0.4474 | 14 |
| 16 | 92 | RaptorX | 38 | 2.3335 | 16 | 0.0614 | 17 | 16.7413 | 13 | 0.4406 | 15 |
| 17 | 77 | FALCON_TOPO | 38 | -4.8168 | 17 | -0.1268 | 20 | 10.311 | 21 | 0.2713 | 22 |
| 18 | 425 | FALCON_TOPOX | 38 | -5.4354 | 18 | -0.143 | 21 | 9.6013 | 23 | 0.2527 | 25 |
| 19 | 16 | FFAS-3D | 38 | -7.5791 | 19 | -0.1994 | 22 | 9.0102 | 24 | 0.2371 | 26 |
| 20 | 464 | tsspred2 | 38 | -7.8335 | 20 | -0.2061 | 23 | 7.4049 | 27 | 0.1949 | 30 |
| 21 | 452 | ZHOU-SPARKS-X | 33 | -9.3695 | 21 | 0.0191 | 18 | 11.5223 | 20 | 0.3492 | 20 |
| 22 | 407 | Distill | 37 | -12.2762 | 22 | -0.2777 | 24 | 6.531 | 33 | 0.1765 | 35 |
| 23 | 258 | MUfold1 | 38 | -13.131 | 23 | -0.3456 | 26 | 7.1229 | 28 | 0.1874 | 31 |
| 24 | 166 | FFAS03 | 36 | -14.9395 | 24 | -0.3039 | 25 | 6.5548 | 32 | 0.1821 | 34 |
| 25 | 359 | Atome2_CBS | 37 | -15.2989 | 25 | -0.3594 | 27 | 6.9284 | 31 | 0.1873 | 32 |
| 26 | 467 | Pareto-server | 36 | -18.0037 | 26 | -0.389 | 28 | 7.0621 | 29 | 0.1962 | 29 |
| 27 | 444 | BhageerathH-Plus | 38 | -18.5619 | 27 | -0.4885 | 30 | 6.9613 | 30 | 0.1832 | 33 |
| 28 | 382 | RBO_Aleph | 38 | -21.1412 | 28 | -0.5563 | 31 | 9.9672 | 22 | 0.2623 | 23 |
| 29 | 28 | M4T-SmotifTF | 34 | -23.8861 | 29 | -0.4672 | 29 | 8.7317 | 25 | 0.2568 | 24 |
| 30 | 357 | FLOUDAS_SERVER | 37 | -25.3194 | 30 | -0.6303 | 32 | 4.8168 | 36 | 0.1302 | 36 |
| 31 | 275 | slbio | 38 | -26.2381 | 31 | -0.6905 | 34 | 8.5587 | 26 | 0.2252 | 27 |
| 32 | 495 | Seok-assembly | 18 | -28.6347 | 32 | 0.6314 | 7 | 11.8328 | 19 | 0.6574 | 9 |
| 33 | 434 | MULTICOM-REFINE | 38 | -31.4382 | 33 | -0.8273 | 36 | 12.1197 | 18 | 0.3189 | 21 |
| 34 | 455 | ACOMPMOD | 35 | -33.5103 | 34 | -0.786 | 35 | 12.3544 | 17 | 0.353 | 19 |
| 35 | 26 | chuo-u2 | 38 | -33.5891 | 35 | -0.8839 | 37 | 2.9636 | 39 | 0.078 | 40 |
| 36 | 380 | chuo-u-server | 38 | -33.5891 | 35 | -0.8839 | 37 | 2.9636 | 39 | 0.078 | 40 |
| 37 | 180 | PhyreTopoAlpha | 38 | -34.1037 | 37 | -0.8975 | 39 | 4.9141 | 35 | 0.1293 | 37 |
| 38 | 421 | MUfold2 | 36 | -38.7313 | 38 | -0.9648 | 40 | 4.0065 | 37 | 0.1113 | 38 |
| 39 | 430 | GOAL_COMPLEX | 11 | -54.8318 | 39 | -0.0756 | 19 | 4.9644 | 34 | 0.4513 | 13 |
| 40 | 451 | RaptorX-Contact | 37 | -55.2342 | 40 | -1.4388 | 41 | 1.1669 | 42 | 0.0315 | 42 |
| 41 | 284 | Seok-naive_assembly | 13 | -58.6469 | 41 | -0.6651 | 33 | 2.6076 | 41 | 0.2006 | 28 |
| 42 | 432 | Pcons-net | 33 | -59.1168 | 42 | -1.4884 | 42 | 3.3478 | 38 | 0.1014 | 39 |
| 43 | 321 | GAPF_LNCC_SERVER | 36 | -59.3802 | 43 | -1.5383 | 43 | 0.504 | 43 | 0.014 | 43 |

Supplementary Table 5. Independent benchmarking or tertiary structure predictions with CASP13 data. “Assessors’ formula” (GDT_HA + (SG + lDDT + CAD)/3 + ASE) data are shown for the server groups on the 'all groups' + 'server only' targets for the TBM + TBM/FM domains. Data are downloaded from <http://predictioncenter.org/casp13/>.

| **#** | **GR code** | **GR name** | **Domains Count** | **SUM Zscore (>-2.0)** | **Rank SUM Zscore (>-2.0)** | **AVG Zscore (>-2.0)** | **Rank AVG Zscore (>-2.0)** | **SUM Zscore (>0.0)** | **Rank SUM Zscore (>0.0)** | **AVG Zscore (>0.0)** | **Rank AVG Zscore (>0.0)** |
| --- | --- | --- | --- | --- | --- | --- | --- | --- | --- | --- | --- |
| 1 | 261 | Zhang-Server | 67 | 58.9148 | 1 | 0.8793 | 1 | 59.4947 | 1 | 0.888 | 1 |
| 2 | 145 | QUARK | 67 | 57.4414 | 2 | 0.8573 | 2 | 57.5857 | 2 | 0.8595 | 2 |
| 3 | 324 | RaptorX-DeepModeller | 67 | 53.2736 | 3 | 0.7951 | 3 | 55.7147 | 3 | 0.8316 | 3 |
| 4 | 221 | RaptorX-TBM | 67 | 49.6283 | 4 | 0.7407 | 4 | 54.088 | 4 | 0.8073 | 4 |
| 5 | 156 | Seok-server | 67 | 49.166 | 5 | 0.7338 | 5 | 51.7771 | 5 | 0.7728 | 5 |
| 6 | 368 | BAKER-ROSETTASERVER | 67 | 39.8337 | 6 | 0.5945 | 6 | 48.9383 | 6 | 0.7304 | 6 |
| **7** | **246** | **IntFOLD5** | **67** | **30.5447** | **9** | **0.4559** | **10** | **41.3513** | **7** | **0.6172** | **7** |
| 8 | 243 | MULTICOM-CONSTRUCT | 67 | 32.4691 | 7 | 0.4846 | 8 | 37.9738 | 8 | 0.5668 | 9 |
| 9 | 164 | Yang-Server | 65 | 28.7508 | 12 | 0.5039 | 7 | 37.5423 | 9 | 0.5776 | 8 |
| 10 | 149 | Zhou-SPOT-3D | 67 | 29.2277 | 11 | 0.4362 | 12 | 37.1025 | 10 | 0.5538 | 10 |
| 11 | 23 | MULTICOM-NOVEL | 67 | 30.7651 | 8 | 0.4592 | 9 | 37.0405 | 11 | 0.5528 | 11 |
| 12 | 58 | MULTICOM_CLUSTER | 67 | 29.2703 | 10 | 0.4369 | 11 | 35.1364 | 12 | 0.5244 | 12 |
| 13 | 441 | FALCON | 67 | -7.622 | 15 | -0.1138 | 19 | 31.3321 | 13 | 0.4676 | 15 |
| 14 | 160 | CMA-align | 64 | 12.9054 | 14 | 0.2954 | 15 | 30.1735 | 14 | 0.4715 | 14 |
| 15 | 498 | RaptorX-Contact | 67 | 19.8215 | 13 | 0.2958 | 14 | 29.9101 | 15 | 0.4464 | 16 |
| 16 | 4 | YASARA | 61 | -8.3438 | 16 | 0.0599 | 17 | 25.5688 | 16 | 0.4192 | 18 |
| 17 | 337 | FALCON-TBM | 67 | -16.6388 | 18 | -0.2483 | 21 | 24.7447 | 17 | 0.3693 | 20 |
| 18 | 347 | MESHI-server | 47 | -20.2989 | 19 | 0.4192 | 13 | 24.2194 | 18 | 0.5153 | 13 |
| 19 | 266 | slbio_server | 58 | -37.8224 | 22 | -0.3418 | 22 | 23.7823 | 19 | 0.41 | 19 |
| 20 | 470 | Seok-assembly | 50 | -28.3917 | 21 | 0.1122 | 16 | 22.1164 | 20 | 0.4423 | 17 |
| 21 | 116 | Zhang-CEthreader | 67 | -25.2285 | 20 | -0.3765 | 23 | 19.8315 | 21 | 0.296 | 22 |
| 22 | 402 | RBO-Aleph | 62 | -37.8772 | 23 | -0.4496 | 24 | 15.6839 | 22 | 0.253 | 23 |
| 23 | 7 | ACOMPMOD | 67 | -47.3156 | 25 | -0.7062 | 26 | 14.3208 | 23 | 0.2137 | 25 |
| 24 | 488 | Delta-Gelly-Server | 65 | -51.3488 | 28 | -0.7284 | 27 | 14.1465 | 24 | 0.2176 | 24 |
| 25 | 432 | Seok-naive_assembly | 39 | -57.2889 | 30 | -0.033 | 18 | 13.3186 | 25 | 0.3415 | 21 |
| 26 | 257 | NOCONTACT | 62 | -63.7308 | 31 | -0.8666 | 32 | 12.8336 | 26 | 0.207 | 26 |
| 27 | 124 | AWSEM-Suite | 66 | -9.6071 | 17 | -0.1153 | 20 | 12.7743 | 27 | 0.1936 | 27 |
| 28 | 110 | Distill | 67 | -49.0642 | 26 | -0.7323 | 28 | 10.2961 | 28 | 0.1537 | 28 |
| 29 | 85 | BhageerathH-Plus | 67 | -55.439 | 29 | -0.8274 | 31 | 9.5977 | 29 | 0.1432 | 29 |
| 30 | 497 | GaussDCA | 66 | -50.6647 | 27 | -0.7373 | 29 | 8.6324 | 30 | 0.1308 | 30 |
| 31 | 312 | MUFold_server | 67 | -65.636 | 32 | -0.9796 | 33 | 7.5792 | 31 | 0.1131 | 31 |
| 32 | 152 | PconsC4 | 66 | -40.986 | 24 | -0.5907 | 25 | 7.3503 | 32 | 0.1114 | 32 |
| 33 | 348 | HMSCasper-Refiner | 67 | -66.0236 | 34 | -0.9854 | 34 | 6.4831 | 33 | 0.0968 | 33 |
| 34 | 407 | rawMSA | 57 | -65.9587 | 33 | -0.8063 | 30 | 4.4812 | 34 | 0.0786 | 34 |
| 35 | 365 | 3D-JIGSAW_SL1 | 67 | -75.9539 | 35 | -1.1336 | 35 | 2.2048 | 35 | 0.0329 | 35 |
| 36 | 41 | FALCON-Contact | 67 | -101.5943 | 37 | -1.5163 | 38 | 1.0372 | 36 | 0.0155 | 36 |
| 37 | 282 | PRAYOG | 60 | -86.2027 | 36 | -1.2034 | 36 | 0.4015 | 37 | 0.0067 | 37 |
| 38 | 458 | FOLDNET | 41 | -103.3451 | 38 | -1.2523 | 37 | 0.2671 | 38 | 0.0065 | 38 |
| 39 | 378 | Cao-server | 67 | -109.1538 | 39 | -1.6292 | 39 | 0.101 | 39 | 0.0015 | 39 |

Supplementary Table 6. Independent benchmarking or tertiary structure predictions with CASP13 data. “Assessors’ formula” (GDT_HA + (SG + lDDT + CAD)/3 + ASE) data are shown for the server groups on the 'all groups' + 'server only' targets for the TBM domains only. Data are downloaded from <http://predictioncenter.org/casp13/>.

| **#** | **GR code** | **GR name** | **Domains Count** | **SUM Zscore (>-2.0)** | **Rank SUM Zscore (>-2.0)** | **AVG Zscore (>-2.0)** | **Rank AVG Zscore (>-2.0)** | **SUM Zscore (>0.0)** | **Rank SUM Zscore (>0.0)** | **AVG Zscore (>0.0)** | **Rank AVG Zscore (>0.0)** |
| --- | --- | --- | --- | --- | --- | --- | --- | --- | --- | --- | --- |
| 1 | 261 | Zhang-Server | 80 | 72.014 | 1 | 0.9002 | 1 | 72.7759 | 1 | 0.9097 | 1 |
| 2 | 145 | QUARK | 80 | 71.0698 | 2 | 0.8884 | 2 | 71.3261 | 2 | 0.8916 | 2 |
| 3 | 324 | RaptorX-DeepModeller | 80 | 65.5509 | 3 | 0.8194 | 3 | 69.4159 | 3 | 0.8677 | 3 |
| 4 | 221 | RaptorX-TBM | 80 | 59.0678 | 4 | 0.7383 | 4 | 64.5294 | 4 | 0.8066 | 4 |
| 5 | 368 | BAKER-ROSETTASERVER | 79 | 50.2939 | 6 | 0.6619 | 5 | 61.879 | 5 | 0.7833 | 5 |
| 6 | 156 | Seok-server | 80 | 51.6225 | 5 | 0.6453 | 6 | 58.319 | 6 | 0.729 | 6 |
| **7** | **246** | **IntFOLD5** | **80** | **36.0524** | **8** | **0.4507** | **9** | **47.4577** | **7** | **0.5932** | **7** |
| 8 | 149 | Zhou-SPOT-3D | 80 | 35.8201 | 9 | 0.4478 | 10 | 45.4796 | 8 | 0.5685 | 8 |
| 9 | 243 | MULTICOM-CONSTRUCT | 80 | 37.0114 | 7 | 0.4626 | 8 | 43.6717 | 9 | 0.5459 | 10 |
| 10 | 164 | Yang-Server | 78 | 33.0273 | 12 | 0.4747 | 7 | 43.5029 | 10 | 0.5577 | 9 |
| 11 | 23 | MULTICOM-NOVEL | 80 | 33.9837 | 11 | 0.4248 | 12 | 41.5072 | 11 | 0.5188 | 11 |
| 12 | 58 | MULTICOM_CLUSTER | 80 | 34.0958 | 10 | 0.4262 | 11 | 40.7894 | 12 | 0.5099 | 12 |
| 13 | 498 | RaptorX-Contact | 80 | 27.1744 | 13 | 0.3397 | 14 | 38.9329 | 13 | 0.4867 | 14 |
| 14 | 441 | FALCON | 80 | -9.8145 | 16 | -0.1227 | 20 | 37.3774 | 14 | 0.4672 | 15 |
| 15 | 160 | CMA-align | 75 | 11.3978 | 14 | 0.2853 | 15 | 33.6927 | 15 | 0.4492 | 16 |
| 16 | 266 | slbio_server | 70 | -46.8297 | 24 | -0.3833 | 23 | 27.428 | 16 | 0.3918 | 18 |
| 17 | 4 | YASARA | 73 | -15.2564 | 17 | -0.0172 | 17 | 27.2182 | 17 | 0.3729 | 19 |
| 18 | 337 | FALCON-TBM | 80 | -21.1199 | 18 | -0.264 | 21 | 27.0144 | 18 | 0.3377 | 20 |
| 19 | 347 | MESHI-server | 53 | -34.8597 | 20 | 0.3611 | 13 | 26.4204 | 19 | 0.4985 | 13 |
| 20 | 470 | Seok-assembly | 56 | -43.1223 | 21 | 0.0871 | 16 | 24.9378 | 20 | 0.4453 | 17 |
| 21 | 116 | Zhang-CEthreader | 80 | -27.7186 | 19 | -0.3465 | 22 | 24.5073 | 21 | 0.3063 | 22 |
| 22 | 124 | AWSEM-Suite | 79 | -6.4237 | 15 | -0.056 | 18 | 19.1578 | 22 | 0.2425 | 24 |
| 23 | 488 | Delta-Gelly-Server | 78 | -58.4129 | 27 | -0.6976 | 27 | 18.6017 | 23 | 0.2385 | 25 |
| 24 | 7 | ACOMPMOD | 80 | -55.595 | 25 | -0.6949 | 26 | 18.2527 | 24 | 0.2282 | 26 |
| 25 | 402 | RBO-Aleph | 74 | -44.5563 | 23 | -0.44 | 24 | 18.067 | 25 | 0.2441 | 23 |
| 26 | 257 | NOCONTACT | 75 | -71.9864 | 30 | -0.8265 | 31 | 16.4022 | 26 | 0.2187 | 27 |
| 27 | 432 | Seok-naive_assembly | 42 | -79.1259 | 33 | -0.0744 | 19 | 13.489 | 27 | 0.3212 | 21 |
| 28 | 110 | Distill | 80 | -61.3512 | 28 | -0.7669 | 30 | 10.9976 | 28 | 0.1375 | 28 |
| 29 | 85 | BhageerathH-Plus | 80 | -67.5328 | 29 | -0.8442 | 32 | 10.1481 | 29 | 0.1269 | 29 |
| 30 | 497 | GaussDCA | 79 | -57.3212 | 26 | -0.7003 | 28 | 9.9215 | 30 | 0.1256 | 30 |
| 31 | 152 | PconsC4 | 79 | -43.8118 | 22 | -0.5293 | 25 | 8.9574 | 31 | 0.1134 | 32 |
| 32 | 312 | MUFold_server | 80 | -81.9114 | 34 | -1.0239 | 34 | 7.8901 | 32 | 0.0986 | 33 |
| 33 | 407 | rawMSA | 69 | -74.0596 | 31 | -0.7545 | 29 | 7.8563 | 33 | 0.1139 | 31 |
| 34 | 348 | HMSCasper-Refiner | 80 | -79.1249 | 32 | -0.9891 | 33 | 6.9776 | 34 | 0.0872 | 34 |
| 35 | 365 | 3D-JIGSAW_SL1 | 80 | -91.6535 | 35 | -1.1457 | 35 | 2.2892 | 35 | 0.0286 | 35 |
| 36 | 41 | FALCON-Contact | 80 | -118.3338 | 37 | -1.4792 | 38 | 1.063 | 36 | 0.0133 | 36 |
| 37 | 282 | PRAYOG | 71 | -100.7148 | 36 | -1.165 | 36 | 0.5434 | 37 | 0.0077 | 37 |
| 38 | 378 | Cao-server | 80 | -126.2643 | 39 | -1.5783 | 39 | 0.4862 | 38 | 0.0061 | 38 |
| 39 | 458 | FOLDNET | 50 | -121.3226 | 38 | -1.2265 | 37 | 0.2785 | 39 | 0.0056 | 39 |

Supplementary Table 7. Independent benchmarking with CASP12 data. CASP12 - McGuffin group performance summary - Analysis on the models designated as "1" All groups on 'all groups' targets. Data downloaded from <http://predictioncenter.org/casp12/>.

| **Scoring metric** | **Target classification** | **McGuffin group ranking (out of 128 groups)** |
| --- | --- | --- |
| Assessors’ formula  (GDT_HA + (SG + lDDT + CAD)/3 + ASE) | TBM + TBM/FM domains | 2nd |
|  | TBM domains | 4th |
| GDT_TS | All domains | 8th |
|  | FM + TBM/FM domains | 8th |
|  | FM domains | 11th |

Supplementary Table 8. Independent benchmarking with CASP13 data. CASP13 - McGuffin group summary - Analysis on the models designated as "1" All groups on 'all groups' targets. Data downloaded from <http://predictioncenter.org/casp13/>.

| **Scoring metric** | **Target classification** | **McGuffin group ranking (out of 98 groups)** |
| --- | --- | --- |
| GDT_TS | All domains | 6th* |
| Assessors' formula  (GDT_HA + (SG + lDDT + CAD)/3 + ASE) | TBM + TBM/FM domains | 6th |
|  | TBM domains | 13th |
| Assessors' formula (GDT_TS + QCS) | FM + TBM/FM domains | 9th |
|  | FM domains | 13th |
| *4th research group, 3rd academic group, 1st academic group outside the US | | |
